# New Kind of Banded Logan Crown

**Published:** 1904-01

**Authors:** A. Almon Babcock

**Affiliations:** Brantford.


### NEW KIND OF BANDED LOGAN CROWN.

By A. Almon Babcock, D. D. S., Brantford.

Read before the Brant County Dental Association.

We will suppose a case presenting with a bicuspid in such condition that filling is impossible, and it is advisable to crown. We will say that the pulp has died a natural death some time previously, and if suppurating at all it is discharging through the crown, and not by fistulæ on the gum.

My first step is to remove all soft deposits in the crown and obtain a good direct opening into the root-canal without cutting off the crown. I believe that in cases where it is possible that it is better to retain the crown of the tooth until the canal is in an aseptic condition, for the following reasons:

1st. If we are unable to get the canal in proper condition for some length of time, the interstitial gum tissue shows an indication to inflame and swell and cover the sides of the root in such a manner as to make it very embarrassing when we do attempt our adaptation, especially if we are going to adopt a Logan.

2nd. We can make use of the frail shell left to hold dressings of loose cotton underneath a temporary stopping, for the purpose of catching and absorbing the discharge from the root-canal. That is, if the pulp is in a suppurative condition, thus keeping the mouth free from the discharge.

3rd. There is not the liability of forcing food into the canal that we dare not tightly close on account of the suppurative gases.

To continue, then, with the treatment. It is to be understood that we have mechanically cleaned the canal by some of our various methods. The next is treatment medicinally for the destruction of germs which would soon tend toward putrefaction if we did not go beyond the mechanical treatment. If the destruction of organic matter has gone so far as to indicate decay of the dentine, it is necessary that the root should be reamed to an extent sufficient (or at least allowable, considering the condition of the root) to remove the softened or contaminated dentine. Then follows the application of our disinfectant, of which there are many to choose from, and the difficulty that I would get into by attempting to show any decided partiality to any one in particular, leads me to simply mention a few of the many, as carbolic acid, nitrate of silver, bichloride of mercury, etc. An application of an antiseptic in the canal until a next sitting is the next step in order.

The essential oils are chiefly used in this stage on account of their adaptability to allaying sensitiveness of the canal that so often is prevalent with the removal of pulps.

Using your own judgment, when the case appears again, as to whether it is in condition to undertake the preparation of trimming for the crowning, the first step is the removal of the crown. With a fine burr, drill a groove around the gum margin of the tooth, and then with the incising forceps the crown can be usually very cleanly cut off.

A facer comes in very handy here, and I think is far ahead of the stone for grinding down, although it is advisable to use the stone afterward. It will trim well around inside of the enamel, and thus you can get beneath the gum-line without injuring the gum; then with enamel cleavers the ring of enamel can be removed and a few applications with properly shaped stones will put the end in proper condition.

Having got this far, I will give my account of making a banded Logan crown for the case, and describe it to the best of my ability.

Trim the root and make the band, and adapt the same as for Richmond. Face the band, being sure that the lingual is as close to the root as possible, this will help you in the matter of space later on.

Next take the crown selected, and make a perfect adaptation to the cap for about one-third of the depth of the crown, labiolingually, then the remaining two-thirds should be sloped off at an angle as acute as the build of the crown (Logan) will admit. This is important, as it is hard to make solder flow in a very acute angle as we all know. Having adapted these two parts to our satisfaction, put a facing over the pin of the Logan, and burnish it over the lingual. This can be easily done by slightly notching the gold along the edge that we want to turn over. This being done, put the cap on the root, and with a small piece of resin wax, or some such compound around the lingual half of the Logan, force the two parts together, holding them there until the compound is hard. Trim off the excess with a warm instrument and remove the whole, invest and solder. This gives a crown that is free from the splitting of facings in the Richmond, with the qualities of the Richmond band in addition.

In bicuspids it does away with the all-gold crown, to which some people object, and puts an all-porcelain one in its place, with a band to protect the cement from the acton of the saliva. As to durability, I can only say that my oldest attempt only dates back to June, 1901, and it is constantly under my supervision, because I can see it every day. Since then I have made quite a number and have lost two. In one I lost the root, and in the other the case came back with the side off, and to this I attributed the fact that the bite was very close, and I had ground the occluding surface considerably to accommodate a lower cuspid, and this weakened it. As to appearance, I think they excel, because the Logan crowns themselves are modelled in excellent patterns of original crowns, and are far ahead of anything we can build ourselves, especially in bicuspids.

